# A Rare Case Report on Neurilemmoma of the Superficial Peroneal Nerve and a Review of the Literature

**DOI:** 10.7759/cureus.24774

**Published:** 2022-05-06

**Authors:** Umesh Yadav, Aditya Seth, Ajay Sheoran, Rahul Kumar, Nishan Yadav, Parul

**Affiliations:** 1 Orthopaedics, Pandit Bhagwat Dayal Sharma Post Graduate Institute of Medical Sciences, Rohtak, IND

**Keywords:** rare cancer, oncosurgery, peripheral schwannoma, nerve root preserving, musculoskeletal tumor

## Abstract

Neurilemmoma is a form of nerve tumor that develops from the nerve sheaths. It is a slow-growing tumor with a rare malignant transformation. It has an incidence rate of less than 1% in lower limbs and its origin in the superficial peroneal nerve is an extremely rare occurrence. In this report, we present a case of neurilemmoma of the superficial peroneal nerve in a 67-year-old male who presented with complaints of pain and swelling at the lateral aspect of the leg. The swelling was enucleated while preserving the main nerve trunk. The patient was found to be asymptomatic after a two-year postoperative period.

## Introduction

A neurilemmoma is a tumor that arises from a single nerve bundle (fascicle) and disrupts the remaining part of the nerve. It may be benign, or malignant in rare cases [1-3]. More fascicles are damaged when a neurilemmoma becomes bigger, making excision more difficult. A neurilemmoma develops over time and if a person develops this tumor, they may notice a painless lump in either the arms or legs. Although neurilemmomas are rarely malignant, they may lead to nerve damage and muscular weakness.

A patient with a suspected pathologic nerve disease is usually evaluated using a combination of physical examination, history, and investigations such as ultrasonography (USG) and MRI. USG can be used for screening a soft tissue mass. MR neurography may reveal a peripheral nerve tumor in more detail, but it is available only at a few centers and is a costly procedure. Additional diagnostic techniques, such as electromyography (EMG) and nerve conduction studies (NCS), measure neuromuscular function to determine if there is denervation, motor unit preservation, or conduction loss. Although neurilemmoma is a rare condition, it has been diagnosed in a variety of locations in the body including the brachial plexus as reported by Lee et al., and it can also affect the legs, ankles, or feet in rare cases [4-6].

## Case presentation

**Figure 1:**
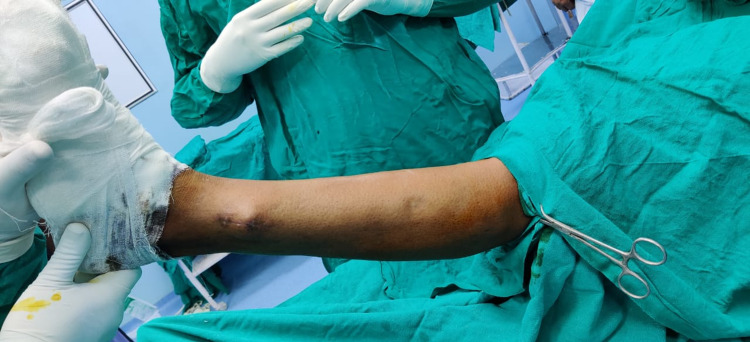
Painful bulge on the lateral aspect of the patient’s left leg along with previous surgical scar mark

A 67-year-old man with no medical history presented to the Orthopaedic Department with a painful bulge on the lateral side of his left leg (Figure 1). He denied any kind of trauma prior to the onset of the symptoms, There was no swelling palpable elsewhere in the body and there was no significant past history or family history. Three years ago, he had discovered this bulge on the lateral side of his left leg, which had been painless. However, six months back, the patient had developed leg pain and noticed that the bulge had been growing slowly. There had been no aggravating or relieving factors linked to it. Initially, the patient had visited a private hospital in a remote area, where an incision had been given under local anesthesia, considering the swelling to be an abscess. However, when the operating surgeon had become doubtful, he had stitched the wound and referred the patient to our institute.

When the patient was examined in the outpatient clinic, a lump measuring 2.3 x 1.9 cm in the subcutaneous plane along with stitched skin margins in the lateral distal portion of the patient's left leg was observed. The patient had moderate tenderness over the lump. On examination of the foot, with both passive and active dorsiflexion, he reported experiencing some discomfort. Deep and superficial peroneal nerve motor functions were both normal. The patient reported mild discomfort and sensory discomfort with active foot inversion (both with and without effort).

**Figure 2:**
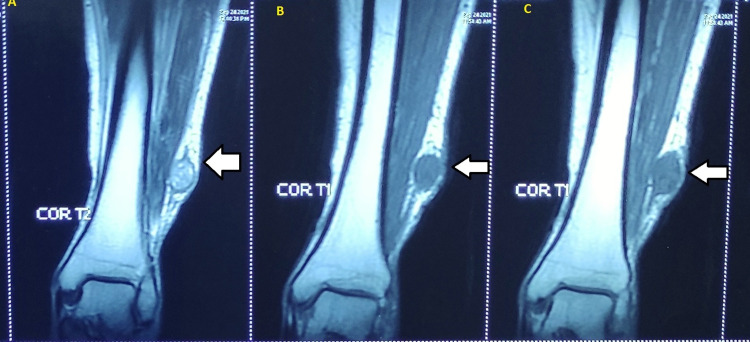
MRI images The images show a lesion measuring approximately 2.3 x 1.9 x 1.5 cm with internal tiny cystic changes seen arising from the left superficial peroneal nerve on the lateral aspect of the left lower leg: (A) T2 images show the hyperintense lesion while hypointensity is seen in T1 images (B, C) MRI: magnetic resonance imaging

MRI (Figure 2) revealed a round-to-oval lesion; a T2 hyperintense lesion measuring approximately 2.3 x 1.9 x 1.5 cm with internal tiny cystic changes was seen arising from the left superficial peroneal nerve on the lateral aspect of the left lower leg in the subcutaneous tissue plane, with mild surrounding edema and without any infiltration into the muscle plane, suggesting a benign peripheral nerve sheath tumor.

**Figure 3:**
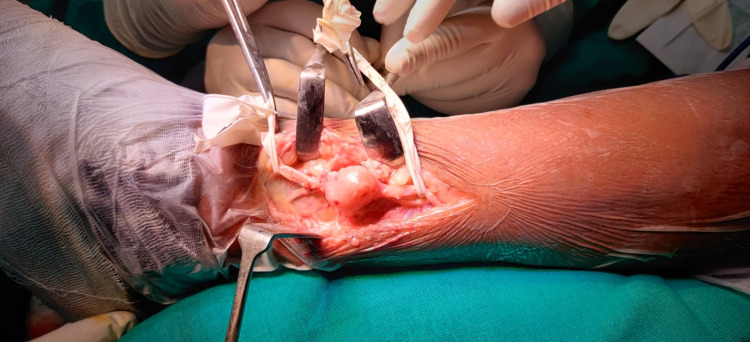
Tumor margins arising from peroneal nerve trunk

**Figure 4:**
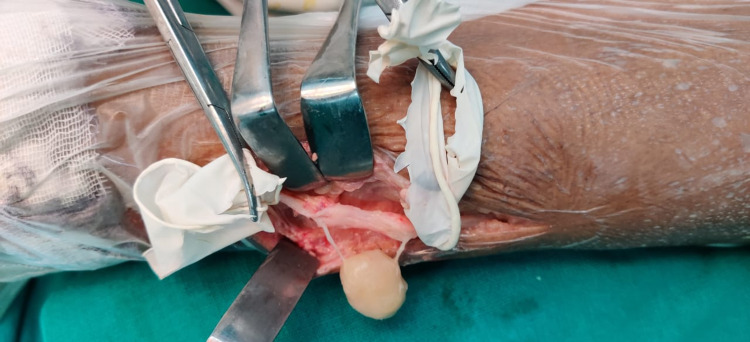
Enucleated tumor mass along single nerve fibers

**Figure 5:**
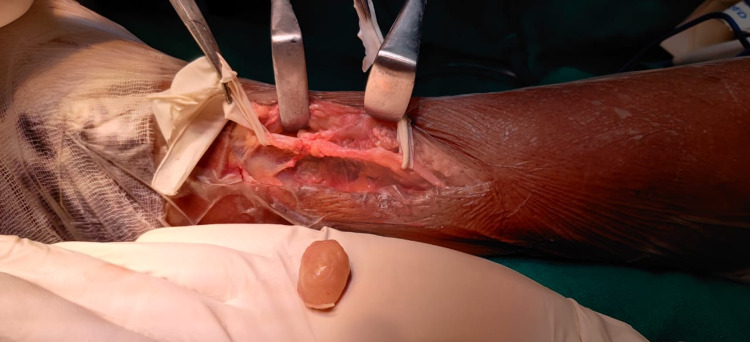
Completely excised tumor mass with preserved nerve root

The patient was anesthetized and a lateral incision was given over the distal fibula; soft tissue was dissected and a tumor was identified arising from the peroneal nerve. Dissection was done around the peroneal nerve and the nerve was separated from the surrounding tissue (Figure 3). The tumor was found to be arising from a single nerve bundle fascicle. An intralesional excision (enucleation) was done and the remaining peroneal nerve was preserved (Figures 4, 5). Histological examination of the removed tumor revealed it to be a benign neurilemmoma.

The postoperative phase went well, and the patient's symptoms significantly improved. He was able to walk unassisted and pain-free.

## Discussion

Neurilemmoma is most commonly found in the age group of 40-50 years, and it predominantly affects females (female-to-male ratio: 1.6:1). It is a rare condition that affects the legs, feet, and ankles, but it is also found in the brachial plexus and sciatic nerve in rare cases [7,8]. Finding the tumor in the peroneal nerve is a rare occurrence, and there is no data available in the literature on the incidence of this occurrence.

Neurilemmomas may appear in a variety of places. They can be asymptomatic, and may also present with mild symptoms or severe symptoms, mostly affecting the nerves. Benign tumors of the tibial and peroneal nerves, especially neurilemmoma, should be investigated in the diagnostic process of non-arthritic and/or non-traumatic toe and foot discomfort.

The diagnosis is based on clinical findings as well as USG and MRI findings. Various signs have been reported in the literature pertaining to peripheral nerve sheath tumors, such as the entering or exiting nerve sign, the target sign, fascicular sign, and split fat sign. However, differentiation between benign and malignant lesions on MRI still remains a challenge [9].

Treatment with excision may relieve symptoms while preserving the function. Neurilemmoma can be solid or cystic in nature, and they have distinct patterns: cellular neurilemmoma have a high cell density and nuclear atypia, but have fewer mitotic figures when compared to malignant neurilemmoma as described by Rodriguez et al. [10,11]. Malignant transformation of a schwannoma (neurilemoma) is an exceedingly rare event. The prognosis for patients with schwannomas undergoing malignant change is poor. No significant signs of malignant transformation were seen in our case at the one-year follow-up.

Since neurilemmomas never traverse through the nerve but remain in the sheath by lying on top of it, they are usually clinically silent. A clear understanding of the nerve structure and the peripheral nervous system can aid in the diagnostic and therapeutic approaches to patients with peripheral nerve pathological conditions. As suggested in the literature, neurilemmoma of cutaneous sensory nerves may be treated with gentle enucleation with a minimal risk of nerve trunk damage as advocated by Lai et al. [8]. However, nerve sheath tumors of motor nerves may require excision, leading to postoperative deficits.

## Conclusions

When encountering cases of lower limb swelling, benign neural sheath neoplasm should always be considered in the differentials, especially in swellings of a non-traumatic origin. Imaging studies, especially MRI, are the most effective investigations for delineating the anatomy and location of the tumor. Enucleation remains the mainstay of treatment while preserving nerve functions.

## References

[1] Rafai MA, El Otmani H, Rafai M (2006). Peroneal nerve schwannoma presenting with a peroneal palsy (Article in French). Rev Neurol (Paris).

[2] Nascimento G, Nomi T, Marques R, Leiria J, Silva C, Periquito J (2015). Ancient Schwannoma of superficial peroneal nerve presenting as intermittent leg pain: a case report. Int J Surg Case Rep.

[3] Dubuisson A, Fissette J, Vivario M, Reznik M, Stevenaert A (1991). A benign tumor of the sciatic nerve: case report and review of the literature. Acta Neurol Belg.

[4] Lee HJ, Kim JH, Rhee SH, Gong HS, Baek GH (2014). Is surgery for brachial plexus schwannomas safe and effective?. Clin Orthop Relat Res.

[5] Tedder JL, Insler HP, Antoine R (1992). Tarsal tunnel syndrome secondary to neurilemmoma. Orthop Rev.

[6] Merritt G 4th, Ramil M, Oxios A, Rushing C (2019). Schwannoma of the plantarmedial aspect of the foot: a case report. Foot (Edinb).

[7] Lee FC, Singh H, Nazarian LN, Ratliff JK (2011). High-resolution ultrasonography in the diagnosis and intraoperative management of peripheral nerve lesions. J Neurosurg.

[8] Lai CS, Chen IC, Lan HC, Lu CT, Yen JH, Song DY, Tang YW (2013). Management of extremity neurilemmomas: clinical series and literature review. Ann Plast Surg.

[9] Kakkar C, Shetty CM, Koteshwara P, Bajpai S (2015). Telltale signs of peripheral neurogenic tumors on magnetic resonance imaging. Indian J Radiol Imaging.

[10] Gainza-Cirauqui ML, Eguía-Del Valle A, Martínez-Conde R, Coca-Meneses JC, Aguirre-Urizar JM (2013). Ancient Schwannoma of the hard palate. An uncommon case report and review. J Clin Exp Dent.

[11] Rodriguez FJ, Folpe AL, Giannini C, Perry A (2012). Pathology of peripheral nerve sheath tumors: diagnostic overview and update on selected diagnostic problems. Acta Neuropathol.

